# Transgenic autoinhibition of p21-activated kinase exacerbates synaptic impairments and fronto-dependent behavioral deficits in an animal model of Alzheimer's disease

**DOI:** 10.18632/aging.101239

**Published:** 2017-05-16

**Authors:** Cyril Bories, Dany Arsenault, Myriam Lemire, Cyntia Tremblay, Yves De Koninck, Frédéric Calon

**Affiliations:** 1 Research Center of Institut Universitaire en Santé Mentale de Québec, Quebec City, QC Canada; 2 Faculty of Pharmacy, Laval University, Quebec City, QC Canada; 3 Centre Hospitalier de l'Université Laval (CHUL) Research Center, Quebec City QC Canada

**Keywords:** Alzheimer, p21-activated kinase, frontal cortex, 3xTg-AD mice, electrophysiology, morphology

## Abstract

Defects in p21-activated kinase (PAK) lead to dendritic spine abnormalities and are sufficient to cause cognition impairment. The decrease in PAK in the brain of Alzheimer's disease (AD) patients is suspected to underlie synaptic and dendritic disturbances associated with its clinical expression, particularly with symptoms related to frontal cortex dysfunction. To investigate the role of PAK combined with Aβ and tau pathologies (3xTg-AD mice) in the frontal cortex, we generated a transgenic model of AD with a deficit in PAK activity (3xTg-AD-dnPAK mice). PAK inactivation had no effect on Aβ40 and Aβ42 levels, but increased the phosphorylation ratio of tau in detergent-insoluble protein fractions in the frontal cortex of 18-month-old heterozygous 3xTg-AD mice. Morphometric analyses of layer II/III pyramidal neurons in the frontal cortex showed that 3xTg-AD-dnPAK neurons exhibited significant dendritic attrition, lower spine density and longer spines compared to NonTg and 3xTg-AD mice. Finally, behavioral assessments revealed that 3xTg-AD-dnPAK mice exhibited pronounced anxious traits and disturbances in social behaviors, reminiscent of fronto-dependent symptoms observed in AD. Our results substantiate a critical role for PAK in the genesis of neuronal abnormalities in the frontal cortex underlying the emergence of psychiatric-like symptoms in AD.

## INTRODUCTION

Alzheimer's disease (AD) is the most common neurodegenerative disorder and the first cause of dementia in the elderly. Alongside memory deficits, patients with AD also display several other behavioral symptoms, including social disinhibition, apathy, anxiety, agitation, and irritability, which are part of the clinical expression of neuropsychiatric symptoms associated with AD [1–3]. Unfortunately, although it is well known that AD neuropathology extends to the frontal cortex [4, 5], the biological substrates of these neuropsychiatric symptoms of AD are still poorly understood. Thus, these symptoms remain very difficult to treat, often increasing an already heavy burden on both patients and caregivers [6].

In recent years, dendritic spine and neuronal dystrophies have been recognized as part of the hallmarks of AD [7–13]. These spine and dendrite abnormalities have a direct impact in synaptic function, intercellular communication and brain function [14, 15], and have also been associated with several mental retardation and psychiatric-like disorders [16]. Both neuropsychiatric diseases and AD have been associated with major metabolic and structural changes of the frontal cortex in humans as well as in animal models [17–20]. We have recently demonstrated fronto-dependent social behavior impairments coinciding with abnormal synaptic function in the frontal cortex in 3xTg-AD mice, a murine model of AD-like amyloidosis and tauopathy [21, 22]. Similarly, deficits in social behavior have been reported in other transgenic models of AD [23–26]. These observations support the underlying role of synaptic defects in the prefrontal cortex in the neuropsychiatric symptoms observed in AD patients [21, 27, 28].

Based on these etiological convergences between AD and psychiatric-like disorders, recent studies have probed common biological substrates. An appealing idea is that AD patients may share common traits at the cellular level with patients harboring developmental mental retardation and psychiatric-like disorders [29, 30]. Consistent with this idea, previous reports demonstrated that PAK1 and 3, both critical regulators of actin and dendritic spine dynamics [31] known to be implicated in mental retardation [32, 33], are down-regulated in AD [9, 10, 34]. Such decrease in PAK has thus been proposed to play a causal role in the loss of dendritic spines and in cognitive symptoms of AD [10, 34, 35]. Indeed, genetically induced inactivation of neocortical PAK activity in transgenic mice reduces the number of dendritic spines and yields to long-term memory deficits [34, 36].

To further define the causal role of PAK defects in AD-like neuropsychiatric symptomatology, we investigated here the role of PAK in an animal model of AD, focusing on molecular endpoints, dendritic morphology and electrophysiology correlates in the frontal cortex. Firstly, we devised a strategy to repress its activity *in vivo*. To this end, we intercrossed a *dnPAK* mouse line, a transgenic model in which the catalytic activity of p21 activated kinase is inhibited [36, 37], with the 3xTg-AD model [22]. To evaluate the consequence of PAK inactivation in the frontal cortex of 3xTg-AD mice, we quantified neuropathological markers of AD, downstream signalization of PAK and synaptic proteins. Single cell morphometric analysis was used to investigate dendritic and spine defects in layer II/III of the prelimbic cortex. At the behavior level, we focused on anxiety and social interactions, which are both regulated by the frontal cortex [21, 38–41] and known to be impaired in AD patients [42–44] and in 3xTg-AD mice [21, 34].

## RESULTS

### Phosphorylated PAK expression is decreased in the frontal cortex of 3xTg-dnPAK mice

**Figure 1:**
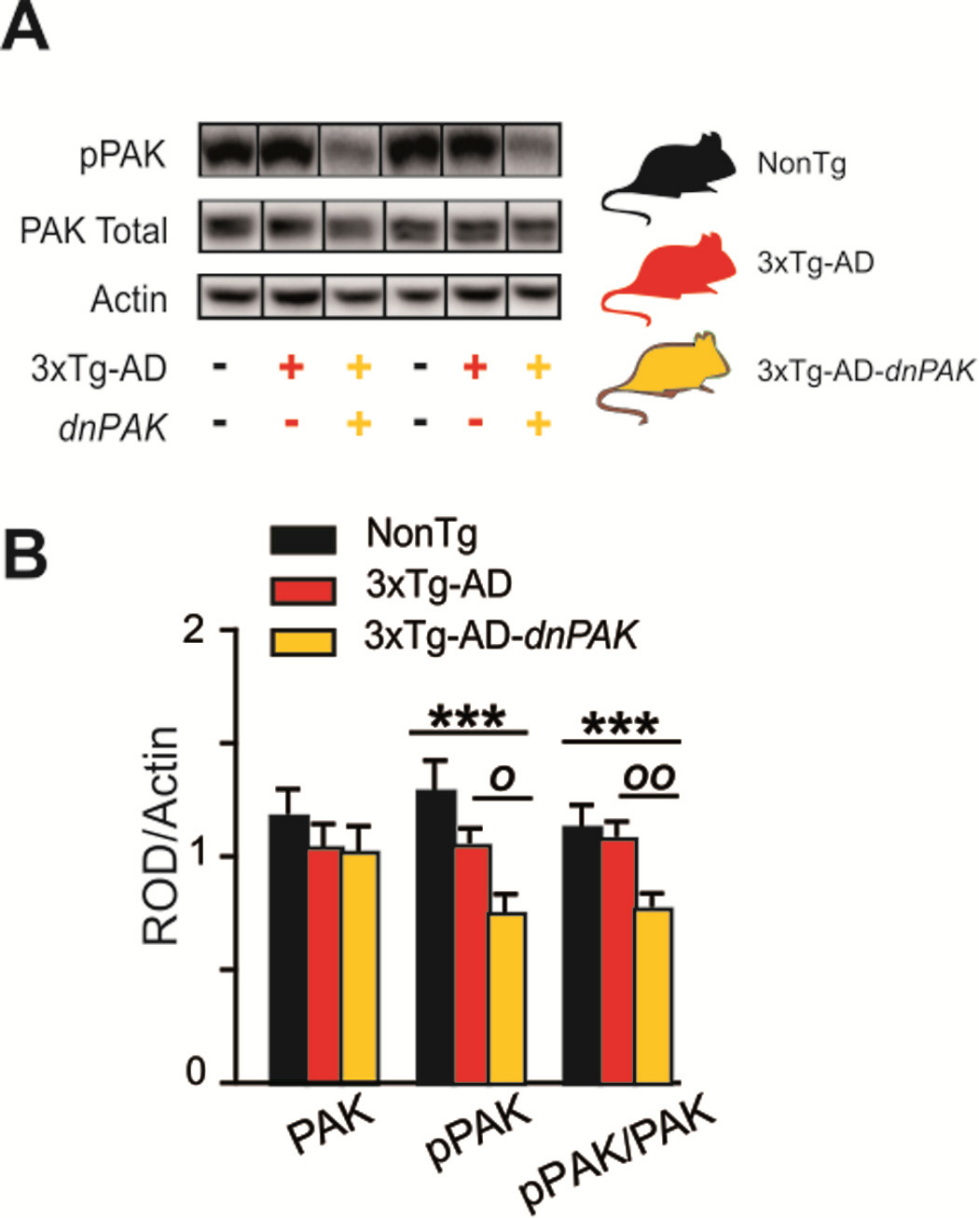
Experimental strategy to generate a mouse model of Alzheimer's disease with a chronic reduction of PAK activity in the forebrain (**A**) *dnPAK* mice were crossed with 3xTg-AD or the corresponding non-transgenic mice (NonTg) yielding three genotypes: NonTg (−/−), 3xTg-AD (+/−) and 3xTg-AD-*dnPAK* (+/−) (**B**). Consistently, phosphorylation of PAK was significantly reduced in the frontal cortex of 18-month-old 3xTg-AD-*dnPAK* animals, confirming the inactivation of PAK. Actin served as an internal control for protein loading (N=12-13 mice for NonTg, N=19-21 mice for 3xTg-AD and N=16-17 mice for 3xTg-AD-*dnPAK*). Examples of Western blots were taken from the same immunoblot experiment for each primary antibody, on the same gel but run in a random order, and rearranged in the same order as the graphs (separated by black lines). *O* p<0.05, *OO* p<0.01, ***p<0.001, Tukey-Kramer post hoc test, *one way* ANOVA. Abbreviations: PAK: p21 activated kinase, *dnPAK:* dominant negative p21-activated kinase, pPAK: phospho-PAK; ROD, relative optical density.

The inactivation of PAK in 18-month-old 3xTg-AD-dnPAK mice was confirmed by the reduction in activated PAK (pPAK phosphorylated at S141) assessed in homogenates from the frontal cortex (Fig. 1). The quantification of pPAK on Western blots revealed a significant decrease in pPAK/PAK ratio in the frontal cortex of 3xTg-AD-dnPAK animals compared to NonTg (Fig. 1B) and heterozygous 3xTg-AD mice (Fig. 1B).

### The inhibition of PAK activity has no effect on Aβ, but increases tau phosphorylation while preventing the pathological increase of insoluble tau in the frontal cortex

**Figure 2:**
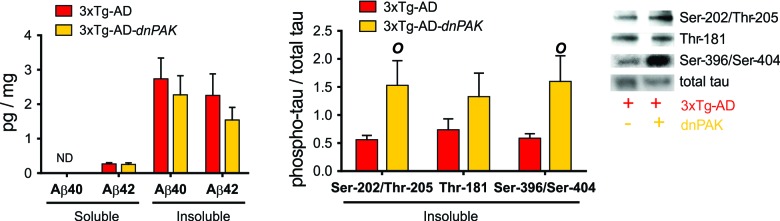
Consequences of the long term inhibition of PAK in 18-month-old 3xTg-AD mice on AD neuropathology markers (**A**) PAK inhibition did not affect Aβ accumulation in frontal cortex, suggesting a downstream position for PAK in Aβ pathological cascade. (**B**) Reduced PAK activity in 3xTg-AD-dnPAK animals led to a higher proportion of tau phosphorylated at Ser202/Thr205 (CP13) and Ser396/Ser404 (AD2) in detergent-insoluble fraction but had no effect on the phospho-epitope Thr181 (AT270). Data expressed as means ± SEM over total human tau (tau-13) (p<0.05, one-way ANOVA, Tukey-Kramer post hoc, *O* p<0.05, student t-test).

We first addressed the consequences of decreased PAK activity on canonical neuropathological markers of AD. ELISA analyses did not reveal any changes in the concentrations of soluble and insoluble Aβ40 and Aβ42 in the frontal cortex of 3xTg-AD-dnPAK animals when compared with the 3xTg-AD group (Fig. 2A). On the other hand, PAK inactivation increased the proportion of tau phosphorylated at Ser202/Thr205 (CP13) and Ser396/Ser404 (AD2) in detergent-insoluble fraction but had no effect on the phosphorylation ratio at Thr181 (AT270) (Fig. 2B). Intriguingly, this rise in phosphorylated tau ratio was mostly driven by a decrease in detergent-insoluble tau as assessed with an antibody raised against human/rodent tau (tau-5) or human tau (tau-13) (Table 1).

**Table 1:** Statistical results

	NonTg Mean+/−SEM	N	3xTg-AD Mean+/−SEM	N	3xTg-AD/dnPAK Mean+/−SEM	N	Statistical test	U, T or F value	p value
**Molecular quantifications (Fig. 1, 2 and 4C)**									
TBS-soluble fraction (cytosolic fraction; ROD/actin)									
tau5	1,19+/−0,14	13	1,02+/−0,07	20	1,07+/−0,07	17	Welch ANOVA	F(2,25.94)=0.5841	p=0.5648
tau 13	-----		1,36+/−0,09	21	1,21+/−0,12	17	Unpaired t-test	T(36)=1.035	p=0.3075
tau CP13	-----		0,97+/−0,08	21	1,14+/−0,11	17	Unpaired t-test	T(36)=1.282	p=0.2079
VGAT	1,35+/−0,1	13	0,92+/−0,08	20**	0,98+/−0,07	17**	One-way ANOVA	F(2,47)=7.486	**p=0.0015**
PAK total	1,2+/−0,1	12	1,06+/−0,09	21	1,04+/−0,1	17	One-way ANOVA	F(2,47)=0.6782	p=0.5124
pPAK (s141)	1,31+/−0,12	12	1,07+/−0,06	20o	0,77+/−0,07	16***	One-way ANOVA	F(2,45)=10.03	**p=0.0002**
pPAK (s141)/PAK total	1,15+/−0,08	12	1,1+/−0,06	19oo	0,79+/−0,05	16***	One-way ANOVA	F(2,44)=9.942	**p=0.0003**
GAD65	1,35+/−0,13	12	0,88+/−0,06	20***	0,91+/−0,07	16**	One-way ANOVA	F(2,45)=9.342	**p=0.0004**
DS-soluble fraction (membranous; ROD/actin)													
synaptophysin	1,06+/−0,1	13	1,04+/−0,07	21	1,01+/−0,05 (17)	17	One-way ANOVA	F(2,48)=0.08795	p=0.9160
PSD95	1,12+/−0,34	13	1,06+/−0,12	21	0,92+/−0,17 (17)	17	Welch ANOVA	F(2,24.817)=0.2841	p=0.7551
GABAa alpha	0,94+/−0,11	13	1,14+/−0,14	21	1,05+/−0,15 (17)	17	One-way ANOVA	F(2,48)=0.4626	p=0.6324
gephyrin	1,07+/−0,11	13	1,01+/−0,05	21	0,98+/−0,09 (17)	17	One-way ANOVA	F(2,48)=0.2993	p=0.7427
VGLUT1	1+/−0,08	13	1,01+/−0,06	21	1,07+/−0,07 (17)	17	One-way ANOVA	F(2,48)=0.2704	p=0.7643
Drebrin	0,97+/−0,19	13	1,01+/−0,72	21	1,04+/−0,16 (17)	17	One-way ANOVA	F(2,40)=1.3420	p=0.2728
Cofilin	1,09+/−0,14	9	1,05+/−0,08	19	0,88+/−0,05 (18)	18	One-way ANOVA	F(2,43)=1.7186	p=0.1914
GluN2B	0,95+/−0,08	13	1,04+/−0,08	21	1+/−0,08 (17)	17	One-way ANOVA	F(2,48)=0.3249	p=0.7242
Formic acid fraction (insoluble fraction; ROD/mg tissu)									
tau CP13/tau 5	-----		0,5515+/−0,0845	14	1,52+/−0,4489	12	Mann Whitney test	U(136,215) = 31.00	**p=0.0069**
AT270/tau5	-----		0,7286+/−0,204	14	1,32+/−0,4262	12	Mann Whitney test	U(168,183) = 63.00	p=0.2917
AD2/tau5	-----		0,5782+/−0,089	14	1,59+/−0,467	10	Mann Whitney test	U(139,161)=34.00	**p=0.0377**
tau 5	0,86+/−0,17	10	1,34+/−0,12	16*o	0,81+/−0,16	12#	One-way ANOVA	F(2,31)=3.568	**p=0.0403**
tau13	-----		1,23+/−0,16	16*o	0,77+/−0,13	15#	Unpaired t-test	T(29)=2.628	**p=0.0136**
tau CP13/tau 13	-----		0,96+/−0,28	15	1,04+/−0,25	12	Unpaired t-test	T(25)=0.2264	p=0.8227
Abeta pathology (pg/mg tissu)									
Soluble-Abeta40	-----		ND ()		ND ()				
Soluble-Abeta42	-----		0,25+/−0,05	17	0,24+/−0,06	10	Unpaired t-test	T(25)=0.1252	p=0.9014
Insoluble-Abeta40	-----		2,72+/−0,62	17	2,26+/−0,57	10	Unpaired t-test	T(25)=0.4989	p=0.6222
Insoluble-Abeta42	-----		2,24+/−0,64	17	1,53+/−0,38	10	Welch t-test	T(23)=0.9539	p=0.3501
Molecular markers (ROD/g proteins)									
TBS-soluble actin	0,89+/−0,07	13	1,03+/−0,07	21	1,04+/−0,07	17	One-way ANOVA	F(2,48)=1.203	p=0.3093
DS-soluble actin	0,94+/−0,07	13	1+/−0,05	21	1,05+/−0,06	17	One-way ANOVA	F(2,48)=0.7679	p=0.4696
**Morphological quantifications (Fig. 3)**									
Spine density (10-60µm)	1,3+/−0,37	9	1,43+/−0,2	15	0,7+/−0,2	11	One-way ANOVA	F(2,32)=1,98	p=0,153
Spine density (60-110µm)	1,46+/−0,27	9	2,22+/−0,38	15	0,9+/−0,16	**10##**	One-way ANOVA	F(2,31)= 4,28	**p=0,022**
Spine density (110-160µm)	1,05+/−0,2	8	2+/−0,4	13	0,4+/−0,1	**8##**	One-way ANOVA	F(2,26)= 5,64	**p=0,0092**
Spine length	1,97+/−0,024	892	2,04+/−0,01	2003	2,12+/−0,03	774***#	One-way ANOVA	F(2,3666)=8,1	**p=0,0003**
Sholl Analysis (length-Basal dendrite)									
10-29	50,54+/−7,55	9	48,68+/−5,78	15	44,96+/−5,58	11	One-way ANOVA	F(2,33)=0.18	p=0.83
30-49	115,65+/−13,01	9	131+/−15,56	15	112,72+/−9,56	11	One-way ANOVA	F(2,33)=0.54	p=0.58
50-69	113,09+/−13,59	9	128,67+/−17,93	15	95,85+/−12,39	11	One-way ANOVA	F(2.33)=1.12	p=0.33
70-89	106,37+/−14,6	8	113,94+/−9,78	15	70+/−9,68	**11#**	One-way ANOVA	F(2,32)=1,98	**p=0.048**
90-109	81,78+/−7,19	8	84,37+/−9,16	14	53,14+/−27,48	10*#	One-way ANOVA	F(2,30)=3.67	**p=0.037**
110-129	49,91+/−7,04	8	53,63+/−7,96	14	42,27+/−7,94	10	One-way ANOVA	F(2,30)=0.55	p=0.583
130-149	22,43+/−4,05	7	22,56+/−4,34	11	20,72+/−6,53	8	One-way ANOVA	F(2,24)=0.04	p=0.96
150-169	10,8+/−2,83	4	11,3+/−10,12	7	16,16+/−7,19	4	One-way ANOVA	F(2,13)=0.67	p=0.52
170-189	8,55+/−1,5	2	10,09+/−1,61	5	8,73+/−2,03	2	NA	NA	NA
190+	8,3+/−NA	1	6,07+/−1,3	3	7,8+/−NA (	1	NA	NA	NA
**Excitatory postsynaptic current (Fig. 4B)**									
Mean frequency (Hz)	1+/−0,39	6	1,44+/−0,39	5	0,58+/−0,18	6	One-way ANOVA	F(2,15)=1,4	p=0,27
Amplitude (pA)	4,44+/−0,37	6	7,89+/−1,6	5 **oo	4,1+/−0,58	**6##**	One-way ANOVA	F(2,15)=4.91	**p=0,024**
**Behavior quantifications (Fig. 5)**									
Exploration (D1)	29,8+/−2,6	13	28,7667+/−1,8	25	25,7+/−2,3576	30	One-way ANOVA	F(2,65)=0.8572	p=0.4291
Exploration (D2)	22,7+/−3,2	13	15,6667+/−1	25	15,9+/−2	30	Welch ANOVA	F(2,26.158)=2.1611	p=0.1353
Exploration (D3)	18,8+/−1,9	13	12,9+/−1.0	25*	13,2+/−1,6	30*	One-way ANOVA	F(2,65)=3.9528	**p=0.0240**
Stand up duration	1,54+/−0,04	16	1,57+/−0,06	24	1,56+/−0,07	21	One-way ANOVA	F(2,58)=0.0774	p=0.9256
Latency	14+/−2,3	16	24,3+/−3,9	26	28,2+/−3,6	20*	One-way ANOVA	F(2,59)=3.4962	**p=0.0367**
Social interaction	-----		166,7+/−16	**15o**	117+/−9	15#	Welch t-test	F(22)=2.674	**p=0.0139**

#p<0.05, ##p<0.01, ###p<0.001 (relative to 3xTg-AD)

o P<0.05, oo P<0.01 (relative to 3xTg-AD/dnPAK)

ROD: relative optical density

ND: not detected

### Pyramidal cell morphology is altered in 3xTg-AD mice with reduced PAK activity

**Figure 3:**
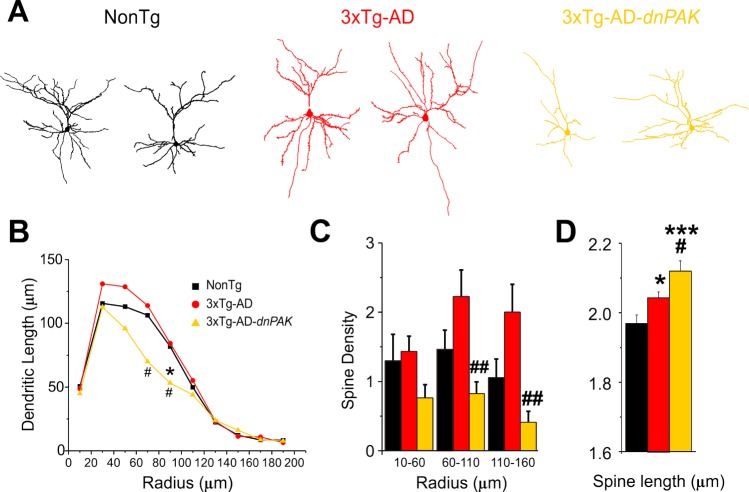
Abnormal dendritic and spine morphologies in 3xTg-AD-*dnPAK* prefrontal cortex (**A**) Examples of reconstructed layer II/III prefrontal pyramidal neurons from NonTg, 3xTg-AD and 3xTg-AD-*dnPAK* animals. (**B**) Sholl analysis of the dendritic length revealed a significant reduction in dendritic arborization 3xTg-AD-*dnPAK* pyramidal cells. (**C**) Spine density in dendrites of layer II/III pyramidal neurons of the prefrontal cortex was significantly lower in 3xTg-AD-*dnPAK* animals. The decrease in spine density was more pronounced in intermediate (60-100 µm from cell body) and distal dendrite segments (110-160 µm), but not significantly different in proximal (10-60 µm) segments. (**D**) Inhibition of PAK activity potentiated the lengthening of spines observed in 3xTg-AD cells when compared with NonTg. # p<0.05 when compared to 3xTg-AD cells, # p<0.05, ## p<0.001 when compared to NonTg cells, one-way ANOVA, Fisher LSD post hoc test. Abbreviations: ROD, relative optical density.

Previous studies report neuronal and spine abnormalities in patients with AD [7–12, 45–48] and in the frontal cortex of 18-month-old hAPP mice, a transgenic mouse model of AD [46]. Structural changes in dendritic spines are also observed in dnPAK mice [36, 37]. Therefore, we investigated for morphological changes of layer II/III pyramidal cells in the medial prefrontal cortex in our transgenic model (Fig. 3A). Sholl analyses revealed a reduction of basal dendrite arborization in 3xTg-AD-dnPAK mice. This effect was mainly observed at a radial distance between 70 and 110 µm (middle range) (Fig. 3B). Moreover, in middle (60-110-µm radius) and distal (110-160-µm radius) dendritic segments, spine density was significantly lower in 3xTg-AD-dnPAK neurons but not in more proximal (10-60-µm radius) dendritic segments (Fig. 3C), compared to 3xTg-AD mice. Finally, our analysis of spine length, calculated as the radial distance from the tip of the spine head to the dendritic shaft [36] revealed that spines from 3xTg-AD neurons were significantly longer when compared with NonTg spines. In addition, lengthy spines in 3xTg-AD animals were further elongated by *in vivo* inhibition of PAK activity (Fig. 3D).

### PAK inhibition alters synaptic properties

**Figure 4:**
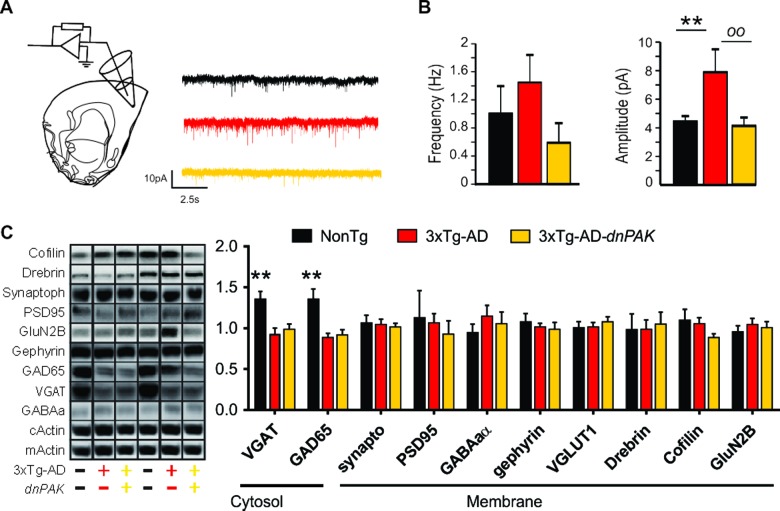
Reducing PAK activity counteracts the enhancement of the glutamatergic synaptic tone observed in 3xTg-AD mice (**A**) Experimental design for patch clamp recordings of layer II/III pyramidal cells of the medial prefrontal cortex. (**B**) While the mean frequency of mEPSCs was comparable between the three genotypes, the mean amplitude of mEPSCs was larger in 3xTg-AD mice. This phenomenon was not present in 3xTg-AD-*dnPAK* mice. (n=5 to 6 cells per group), **p<0.01, ^oo^p<0.01, Fisher LSD post hoc test (**C** and **D**). A significant reduction in GAD65 and VGAT expression was observed in both 3xTg-AD and 3xTg-AD-*dnPAK* animals when compared with NonTg animals (N=12-13 mice for NonTg, N=19-21 mice for 3xTg-AD and N=16-17 mice for 3xTg-AD-*dnPAK*). Examples of Western blots were taken from the same immunoblot experiment for each primary antibody, on the same gel but run in a random order, and rearranged in the same order as the graphs (separated by black lines). **p<0.01, *one way* ANOVA Tukey-Kramer post hoc test.

To assess the functional consequences of the chronic inhibition of PAK activity, we performed patch clamp recordings of the synaptic activity impinging on layer II/III pyramidal neurons in the medial prefrontal cortex. Glutamate receptor-mediated mEPSC activity was recorded at a holding potential of −60 mV. The mean frequency recorded was not significantly different between groups. Our analysis revealed higher mEPSC amplitude in 3xTg-AD mice, a change not observed in 3xTg-AD-dnPAK animals compared to NonTg controls (Fig. 4A and B).

Next, we investigated whether synaptic changes at the functional and structural levels were echoed at the molecular level. We did not observe any significant changes in the expression of drebrin or cofilin (Fig. 4C), two proteins regulated by PAK and known to be altered in AD [10, 34, 49]. However, we observed a significant reduction in the expression of GABA-related presynaptic proteins, GAD65 and VGAT, in both 3xTg-AD and 3xTg-AD-dnPAK groups when compared with NonTg mice, whereas specific markers of glutamatergic synapses (PSD95, GluN2B and Synaptophysin) and postsynaptic markers of GABAergic synapses (Gephyrin, GABA_A_ receptor) remained unchanged (Fig. 4C). In sum, levels of postsynaptic or presynaptic proteins were not associated with functional impairments of excitatory synapses whereas the expression of AD transgenes led to a reduction of GABAergic presynaptic proteins in the frontal cortex.

### PAK inhibition induces anxiety-like behavior and impairs social interaction activity

**Figure 5:**
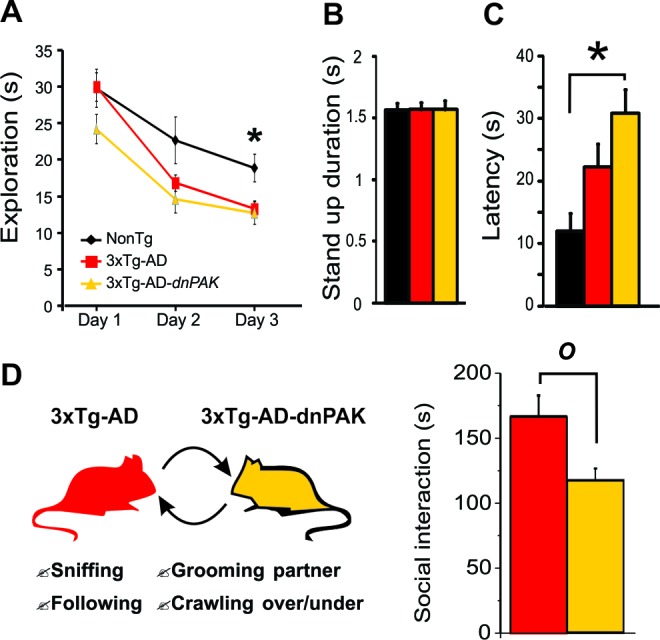
PAK inhibition aggravates the behavioral alterations observed in 3xTg-AD mice (**A**) Both 3xTg-AD and 3xTg-AD-*dnPAK* displayed a reduced exploratory activity in a hole-board task. *p<0.05 when compared to 3xTg-AD and 3xTg-AD-dnPAK animals. (**B**) No significant difference in mean stand-up duration. (**C**) 3xTg-AD-*dnPAK* mice displayed increased anxiety in dark and light box testing as shown by the greater latency time to go out from the dark box. *p<0.05(**D**) Schematic representation of social interaction paradigm used to assess behavioral performance of 3xTg-AD and 3xTg-AD-*dnPAK* animals. Mice with genetically reduced PAK activity interacted less than 3xTg-AD animals during a social interaction test. *O* p<0.05, n=8 animals per group.

To test whether the behavioral performances of the animals were affected by the chronic autoinhibition of PAK activity, a series of evaluations were conducted. First, exploratory behavior was documented using a hole-board task for three consecutive days. Both 3xTg-AD and 3xTg-AD-dnPAK animals displayed reduced level of activity on the third day compared to NonTg animals (Fig. 5A) revealing a decreased motivation to explore their new environment. These observations were independent of changes of basic motor function, as shown by the lack of difference of mean stand-up durations between the different groups of animals (Fig. 5B). Second, we tested whether 3xTg-AD-dnPAK mice were more anxious. To this end, we exposed the mice to the light/dark box test, and we observed that 3xTg-AD-dnPAK mice displayed a higher latency to go out from the dark compartment when compared with NonTg animals (Fig. 5C). This finding suggests that PAK inhibition exacerbated anxious behavior in 3xTg-AD mice. Finally, we tested for the specific impact of PAK inhibition onto social interaction activity, known to be disrupted in 3xTg-AD mice [21, 34]. When compared with 3xTg-AD animals, 3xTg-AD-dnPAK mice displayed a significant reduction in their social interaction activity (Fig. 5D), suggesting defects in fronto-dependent social behaviors caused by the inactivation of PAK.

## DISCUSSION

Besides Aβ deposition and tangle formation, intracellular signaling pathways involved in the pathogenesis of AD are subjects of intense scrutiny as potential drug targets. More particularly, cell pathways regulating dendritic and synaptic organization attract considerable attention. As such, the PAK1-3-related cascades are of particular interest due to their potential causal role in cognitive deficits in AD, but also in mental retardation [10, 29, 45, 50–52]. To further evaluate the role of PAK in AD, we generated a 3xTg-AD mouse with a constitutive reduction of PAK activity. Using this experimental approach, we investigated the consequences of PAK inactivation in the frontal cortex of 3xTg-AD mice at the molecular, synaptic and cellular levels, as well as on fronto-dependent behaviors reminiscent of AD neuropsychiatric symptoms.

### PAK inactivation did not promote total Aβ or tau aggregation, but increased the phosphorylation ratio of insoluble tau

The 3xTg-AD mouse provides the opportunity to investigate the effect of PAK deficit on neuropathological hallmarks of AD, Aβ and tau pathologies. Firstly, the present results are consistent with a downstream position for PAK in the β amyloid cascade. Indeed, PAK inactivation had no effect on Aβ levels. In contrast, previous report emphasized a downregulation of PAK after exposure to Aβ *in vitro* or in the brain cortex or hippocampus of transgenic models of AD [10, 34, 35]. Considering the effect of PAK on tau neuropathology, our results rather suggest an upstream position for PAK1-3 in the molecular pathway controlling the phosphorylation and the accumulation of insoluble tau in 3xTg-AD-dnPAK animals. Indeed, PAK inactivation enhanced the proportion of tau phosphorylated at Ser202/Thr205 (CP13) and Ser396/Ser404 (AD2), but not at the Thr181 (AT270) phospho-epitope. Interestingly, deficits in prepulse inhibition of acoustic startle are driven by tau phosphorylation at Ser396/404 and Ser202 in insoluble protein fractions of female rTg(tauP301L)4510 mice [53]. In addition, tau phosphorylation at Thr205 may regulate the trans-synaptic transport of tau, a mechanism suspected to spread tau pathology in the brain [54]. In keeping with our present observations, elevated Ser396 phospho-tau in the frontal cortex has been found to be associated with neuropsychiatric behaviors in AD patients [55]. Since hyperphosphorylation of insoluble tau at Ser396/Ser404 is a major correlate of impaired cognition in AD [56], increased PAK activity could provide a means to improve tau phosphorylation status and AD symptoms.

### Inactivation of PAK worsens dendrite atrophy and loss of spine in 3xTg-AD mice, two neurophysiological markers of AD

Our single-cell labeling analysis revealed an attrition of the basal dendritic arbor and a reduction in spine density in 3xTg-AD-dnPAK cells, whereas no major differences in these parameters were observed between hemizygous 3xTg-AD and NonTg neurons. Since a reduction of spine density was reported in the frontal cortex layer III neurons of homozygous 3xTg-AD [57], the expression degree of tau and Aß is likely to be an important factor to induce loss of synaptic spine. Our results also suggest that a repression of PAK activity precipitates the atrophy of the dendritic tree, as observed in late stage of AD [58–61]. The lack of dendritic morphological changes in 3xTg-AD mice may result from a transient increase in synaptic density or dendritic lengthening as observed in the neocortex during presymptomatic and early stages of AD [7, 51, 58, 62–64] which may also explain the variability observed in our data. It is also known that an increase in synapse density is thought to precede morphological alterations associated with Aβ and tau-immunoreactive neuritic plaques [7, 8, 57, 62–66]. Therefore, our results suggest that 3xTg-AD animals model an early stage of the disease [67], in which inhibiting PAK activity would accelerate synaptic decline, spine loss and dendritic atrophy, as generally observed in advanced phases of AD [8, 10, 29, 45, 49, 68, 69].

### Spine morphometric abnormalities are aggravated by PAK inactivation in 3xTg-AD mice

We observed a lengthening of spines in 3xTg-AD-dnPAK cells, an exacerbation of a phenotype already present to a lesser degree in 3xTg-AD cells. Thinner and longer spines are generally associated with an immature state, a phenomenon suspected to underlie several neurological and psychiatric disorders [16, 36, 37]. Such changes in spine morphology have also been described in AD [29, 70]. In the brain of rodents or in vitro, exposure to Aβ oligomers leads to a reduction in stubby and mushroom spines found in healthy neurons and is associated with the transient formation of aberrant spines with filopodial or large, branched morphologies reminiscent of structures observed in mental retardation [11, 12, 71]. Nevertheless, our data contrast with previous description of shorter and larger spines in the cortex of dnPAK transgenic animals when compared with non-transgenics [36, 37]. This further suggests that the function of PAK might be profoundly modulated by Aβ/tau pathology in the 3xTg-AD mice, resulting in a switch in the function of PAK. The translocalization of PAK from cytosol to membrane in Tg2576 transgenic mice supports this hypothetical functional switch in an AD-like neuroenvironment [9]. Although interactions between neuropathological markers of AD and PAK function remain poorly understood, our results suggest a key role for PAK in spine morphometric abnormalities observed in AD.

### PAK inactivation prevents functional impairments of glutamatergic synapses: a consequence of the effects of PAK on aggregated tau, spine density and dendrite atrophy?

An important question to investigate was whether these morphological alterations translated into functional changes at the synaptic level. Our electrophysiological recordings revealed that the synaptic activity mediated by AMPA receptors was significantly enhanced in 3xTg-AD compared to NonTg cells. The finding of a greater glutamatergic signaling in 3xTg-AD cells is consistent with the postulated redistribution of abnormally hyperphosphorylated tau to the somatodendritic compartment during AD pathogenesis, boosting excitotoxic signaling and increasing the toxic effects of Aβ [71]. In addition, the lower mEPSC amplitude in 3xTg-AD-dnPAK neurons (compared to 3xTg-AD) is in accordance with the reduction of spine density and the altered dendritic arborization in these neurons, suggesting a decrease in the number of synaptic contacts. Taken together, these results further indicate that 3xTg-AD neurons display neurophysiological defects (upregulation of glutamatergic function) seen early in the disease [62, 63, 67], while the combination with chronic PAK underactivity generates an animal model with synaptic features closer to advanced AD (dendrite atrophy, loss of spine density, more pronounced spine elongation and excitatory synaptic function) [72–78].

Surprisingly, the effect of 3xTg-AD transgenes expression on EPSC in frontal cortex differed with previous observations in the entorhinal cortex [34], in which a higher frequency of EPSC was recorded without any change in the mean amplitude. This contrast could be explained by a difference in the nature of the detected activity. Indeed, the previously measured synaptic activity in the entorhinal cortex was dependent of action potential (without TTX) [34] whereas here we detected pure synaptic activities (with TTX, independent of action potential) in the frontal cortex. The synaptic activity recorded in entorhinal cortex is more representative of basal brain activity since it includes activity related to neuronal activity (dependent of action potential) whereas the pure synaptic activity investigated in this study is a better parameter to focus only in synaptic function. In other words, both approaches dependent of different factors and do not provide the same information. Consequently, both results cannot be directly compared.

### Transgene expression reduced presynaptic markers of GABAergic synapses but did not influence the level of drebrin/cofilin

In the last decade, studies reported neurological deregulations potentially involved in the pathophysiology or symptomatology of AD. The GABA system is one of them. The downregulation of key proteins related to the GABAergic system in the frontal, temporal and parietal cortices of AD patients is consistent with the view that GABAergic function plays a role in AD [79, 80]. In addition, the memory/learning deficits in mice model of AD correlated with astrocytic GABA dysfunction and pharmacological correction of this alteration improves memory capability [81]. In agreement with this GABAergic hypothesis, we found lower levels of two GABAergic presynaptic markers (GAD65 and VGAT) in the frontal cortex of 3xTg-AD mice. Inactivation of PAK activity in transgenic mice did not influence these two molecular impairments, suggesting that PAK is not involved in GABAergic changes in frontal cortex of 3xTg-AD mice. In parallel, it has been proposed that PAK dysfunction leads to cognitive impairment through drebrin displacement and development of cofilin-positive Hirano bodies, both disrupting the actin scaffold [9, 10, 34]. This hypothesis stems from repeated demonstration of a drebrin loss in the brain of AD patients [7, 49, 82]. Our quantification did not reveal significant changes in the levels of drebrin and cofilin in the frontal cortex with transgene expression, suggesting that the effect of PAK might pertain more to activity, subcellular localization or protein interactions of drebrin and cofilin, rather than their total concentrations, at least in the frontal cortex.

### PAK inactivation exacerbates anxiety-like behavior and social apathy, reminiscent of neuropsychiatric symptoms observed in AD

The worsening of anxiety and social behavior induced by the inactivation of PAK is one of the most striking observations reported here. Besides memory deficits, a number of less well-characterized but frequent behavioral symptoms are part of the clinical expression of AD [83, 84]. Among these psychiatric symptoms, anxiety, alterations of personality and social interaction are particularly distressful. However, despite recent advances [21, 23, 34, 85–89], these symptoms are still poorly characterized in animal models of AD, and their neurobiological substrates remain poorly understood. Previous reports demonstrated that impaired cortical activity and abnormal neuronal morphology in the medial prefrontal cortex may contribute to the emergence of emotional and social interaction disturbances [20, 90, 91]. This suggests that the structural and functional abnormalities induced by PAK inactivation in the frontal cortex of 3xTg-AD mice are involved in AD-associated social changes and support idea that the 3xTg-AD-dnPAK mouse is a model of advanced AD. In addition, the higher level of anxiety observed in 3xTg-AD-dnPAK mice is in agreement with the most important morphometric abnormalities observed in frontal cortex, a cerebral structure involved in the circuitry of anxiety [92, 93]. In conclusion, the role of PAK on neuronal morphology and on the fronto-dependent behavioral alterations in AD conditions demonstrated the potential of this protein as a therapeutic target in the treatment of AD-related psychiatric symptoms.

## CONCLUSION

Overall, our results indicate that repressing PAK activation aggravates anxious behavior and impairs social behavior in the 3xTg-AD mouse. These behavioral abnormalities were not coupled to changes in Aβ or excitatory synaptic markers in the frontal cortex. On the other hand, PAK inactivation induced dendritic reduction, aggravated the AD-related spine elongation and increased the relative phosphorylation of insoluble tau at Ser202/Thr205 and Ser396/Ser404 in the frontal cortex, which may be involved in the behavioral impairments observed. As such, PAK inactivation in the 3xTg-AD mouse generated or exacerbated de novo key AD-like signs and symptoms, i.e. dendritic spine defects, anxiety and social interaction deficits. Therefore, preventing PAK inactivation in the frontal cortex may prove to be a valid approach to treat neuropsychiatric-like symptoms of AD.

## MATERIALS AND METHODS

### Animals

Animals were produced and maintained in the animal facilities of the CHUL Research Center at 22 ± 1°C under a 12-h light/dark cycle regime. Water and food were available ad libitum. All experiments were approved by the Laval University Animal Care and Use Committee in accordance with the standards of the Canadian Council on Animal Care.

### Transgenic mice

Original breeders couples of homozygous 3xTg-AD mice, non-transgenic littermates and hemizygous dnPAK transgenic mice were kindly provided by Dr. Laferla [22, 94] and Dr. Tonegawa [36], respectively, to start our own colonies. The 3xTg-AD mouse line was produced from the cointegration of the APP and tau transgenes in the same genetic locus, into single-cell embryos from homozygous PS1-knockin mice, generating mice with the same genetic background. Non-transgenic (NonTg) mice used here are littermates from the original PS1-knockin mice and are on the same background as 3xTg-AD mice (C57BL6/129SvJ) [22]. In the dnPAK mouse line, the mouse Camk2a promoter directs postnatal forebrain expression of the mouse dominant negative autoinhibitory domain (AID) of the p21 protein (Cdc42/Rac)-activated kinase (Pak) gene. The dnPAK transgene was purified and microinjected into C57BL6 zygotes to generate transgenic mice, as described [36, 37]. The dnPAK line was intercrossed in our facilities with 3xTg-AD and their corresponding NonTg control animals. Three groups were generated: a non-transgenic group (NonTg), a hemizygous triple transgenic model of AD group (3xTg-AD) and, finally, a hemizygous triple transgenic mouse model of AD with reduced PAK activity (3xTg-AD-dnPAK) (Fig. 1A), all on the same genetic background [34]. Over one hundred mice were produced and the 3 groups were constituted according to genotype. All experiments were performed on animals between 18 and 20 months of age (18.1 ± 1 months, mean ± S.D), with an equivalent number of males (n=51) and females (n=59) per group.

### ELISA and Western immunoblotting

To collect molecular endpoints, animals were perfused with 10 mM phosphate buffered saline (PBS) containing a cocktail of protease inhibitors (SIGMAFAST™, Sigma–Aldrich, St. Louis, MO) along with phosphatase inhibitors (50 mM sodium fluoride and 1 mM sodium pyrophosphate). Frozen extracts of the frontal cortex were dissected and kept at −80°C. Homogenates from cytosol (TBS-soluble), membrane (detergent-soluble) and detergent-insoluble (formic acid–soluble) fractions were generated for ELISAs and Western immunoblotting analyses as described (Arsenault et al., 2011). Insoluble and soluble Aβ40 and Aβ42 were measured using Human β-Amyloid (1-42) and (1-40) ELISA kit High Sensitive (WAKO, Osaka, Japan) as described [56, 95]. Protein concentrations in samples were determined using bicinchoninic acid assays (Pierce, Rockford, IL) and equal amounts of protein per sample (15 µg of total protein per lane) were added to Laemmli's loading buffer, heated to 95°C for 5 min before loading, and subjected to sodium dodecyl sulfate-polyacrylamide gel electrophoresis. Proteins were electroblotted onto PVDF membranes (Millipore, MA) before blocking in 5% nonfat dry milk and 1% bovine serum albumin (BSA) in PBS containing 0.1% of tween-20 for 1 h. Membranes were immunoblotted with appropriate primary and secondary antibodies followed by chemiluminescence reagents (KPL, Gaithersburg, MD). Band intensities were quantified using a KODAK Image Station 4000 Digital Imaging System (Molecular Imaging Software version, KODAK, New Haven, CT).

The following primary antibodies were used in Western immunoblotting experiments: rabbit anti-vesicular GABA transporter (VGAT; Novus Biologicals, #NB110-55238), mouse anti-PSD-95 (NeuroMab, #75-028), mouse anti-synaptophysin (Millipore, #MAB332), mouse anti-actin (ABM, #Y061021), mouse anti-tau 5 (Millipore, clone tau 5, #MAB361), mouse anti-tau (Covance, clone tau-13, #MMS-520R-500), mouse anti-tau CP13 (phosphorylated at Ser202/Thr205, 1:1000, gift from Dr Peter Davies, Albert Einstein College of Medicine, New York, NY), rabbit anti-cofilin (Cell signaling technology, #3312L), mouse anti-drebrin (Progen Biotechnik GmbH, #GP254), rabbit anti-total PAK1/2/3 (Cell signaling technology, #4750S), rabbit anti-phospho PAK1/2/3: pS141 (BioSource, #44940G), rabbit anti-gephyrin (Abcam, #ab25784), rabbit anti-GAD65 (Millipore, #ABN101), mouse anti GABA_A_ receptor subunit 1 (Neuromab, 1:250, #75-136) and mouse anti-GluN2B (Covance, clone n59/36, #MMS-5148-100).

### Morphometric analyses

Freshly sliced and fixed 250-µm-thick slices taken from 4% PFA were used to inject neurons with Lucifer Yellow (LY) (Lucifer Yellow-Lithium Salt, Invitrogen, L12926) as described previously [96]. Briefly, pyramidal neurons in layer II/III of the prelimbic area of the frontal cortex were impaled with a micropipette containing 1% LY in phosphate buffer (PB) 0.2 M and injected at 0.5-2 nA for approximately 5-10 min to fill the dendritic tree until spines on apical tuft dendrites became clearly visible. For revelation, slices were rinsed in PB 0.1 M three times for 10 min and incubated overnight at 4°C in a solution of PB 0.1 M, sucrose 5% and Triton 0.1%. On the second day three washes of 10 min in PB 0.1 M and 0.1% Triton were performed before incubating in a quenching solution (PB 0.1 M, 0.1% Triton, 0.3% H_2_O_2_) 2 h at room temperature (RT°). Slices were washed three times during 10 min in PB 0.1 M and 0.1% Triton. After 2 h at RT° in a preblocking solution (PB 0.1 M, 0.1% Triton, 10% normal goat serum, NGS), slices were incubated overnight at 4°C with a rabbit anti-LY primary antibody (ThermoFisher) # A-5750) in PB 0.1 M, 0.1% Triton, 5% NGS. On the third day, slices were incubated in a goat anti-rabbit biotinylated secondary antibody (Vector Laboratories, BA-1000) for 2 h at RT°, in PB 0.1 M, after three washes of 10 min each in PB 0.1 M. Slices were finally rinsed with three other 10-min washes in PB 0.1 M. The reaction solution was made from an ABC kit (Vectastain by Vector Laboratories, PK-6100). Sections were washed again three times for 10 min in PB 0.1 M. A 3,3´-diaminodbenzidine (DAB, Sigma-Aldrich #D5905)-Nickel 0.025% solution was filtered and applied to slices for 15 min. Then, 0.006% H_2_O_2_ was added to the well and slices were removed from the solution few seconds after and washed several times in PB 0.1 M to stop the reaction. We thus obtained long-term photostable labeling of dendritic spines and fine neuronal processes. Neurons were reconstructed and spines marked using an Olympus microscope equipped with a 63x objective (1.3 NA), a motorized stage, video camera system, and Neurolucida morphometry software (MBF Bioscience) by an experimenter blind to the experimental conditions. Only clear spines with visible neck and head were marked. Dendritic length and Sholl analysis were obtained for each neuron with NeuroExplorer software (MBF Bioscience). Only neurons with a clear pyramidal shape were kept for further analyses. Additionally, analyses and results presented were restricted to basal dendrites, because several pyramidal cells exhibited various shapes of apical dendrites (single, bitufted) displaying unclear terminal points (real vs. cut during slicing).

### Electrophysiology

#### Subjects

None of the animals used in this study presented any evidence of tumors. A total of 27 mice were used for electrophysiological recordings. 3xTg-AD mice and NonTg were dispatched into three groups according to genotype.

#### Slice preparation and electrophysiology

Mice were deeply anaesthetized with ketamine and xylazine and then decapitated. The brain was removed quickly and placed in ice-cold solution containing (in mM) 210 sucrose, 3.0 KCl, 0.75 CaCl2, 3.0 MgSO4, 1.0 NaH2PO4, 26 NaHCO3, and 10 glucose, saturated with 95% O2 and 5% CO2. Coronal slices of frontal cortex including the prelimbic area were 250 µm thick and kept in artificial cerebral spinal fluid (ACSF) containing (in mM) 124 NaCl, 3.0 KCl, 1.5 CaCl2, 1.3 MgSO4, 1.0 NaH2PO4, 26 NaHCO3, and 20 glucose, gassed with 95% O2-5% CO2 at RT°. Slices were allowed to recover for at least 1 h before recording. A slice was then transferred to a chamber exposed to ACSF flowing at a rate of 2-3 ml/min. Recordings were performed between 32°C-34°C.

#### Whole-cell patch clamp recording

Patch pipettes (6-8 MΩ) were pulled from borosilicate glass capillaries (World Precision Instruments) and filled with an intracellular solution (pH 7.2; 275–280 mOsm) composed of (in mM): 100 Cs methyl sulfonate, 5 CsCl, 10 HEPES, 2 MgCl2, 1 CaCl2, 11 BAPTA, 4 ATP, 0.4 GTP, and 0.1% Neurobiotin Tracer (Vector Laboratories, SP-1120), 0.1% Lucifer Yellow. The junction potential of the pipette was corrected by subtracting 12 mV from recorded membrane voltages. A Multiclamp 700B amplifier (Axon Instruments) was used for the recording. The access resistance was monitored throughout each experiment and only recordings with stable access were used. Experiments were conducted using the Clampex program (pClamp 9.2, Axon Instruments), data collected were 3-kHz filtered and digitized at 10 kHz. The extracellular concentration of potassium was raised to 5 mM to increase the frequency of postsynaptic currents (PSCs) [96]. Tetrodotoxin (TTX; 1 μM, Alomone Labs, T-500) was added to the ACSF to block voltage-gated sodium channels and isolate action potential-independent miniature postsynaptic currents (mPSCs). Whole-cell patch clamp recordings were performed in voltage-clamp mode while maintaining the membrane potential at the reversal potential for GABAA receptor-mediated PSCs (-60 mV) to isolate miniature excitatory postsynaptic currents (mEPSCs). To avoid any long-term effect of the application of TTX to the whole bath, only one cell per slice was recorded. For purpose of analysis, data were filtered at 1 kHz. The Clampfit 9.2 and Origin 8 (OriginLab) software were used for analyses.

### Hole-board exploration test

Exploratory behavior of mice was assessed in a rectangular arena (40 cm X 22 cm) with holes in each corner. Hole-board exploration is a behavioral paradigm that is used frequently in mice and requires no training [34]. The time spent in active exploration of the four holes was measured during a single 5 min daily session for three consecutive days.

### Dark-light box test

Anxiety-like behavior was assessed for 10 min using a two-compartment box (dark and light box). Animals were positioned in the dark compartment and the latency to enter in the light compartment was measured. Additionally, the number of standings and their average duration was measured as a control for locomotor integrity [34, 97, 98].

### Social interaction paradigm

Animals were submitted to a social interaction test performed in a transparent plastic arena (40 cm x 22 cm x 18 cm). Pairs of age-matched animals, unfamiliar with each other, were placed in the unfamiliar test arena for an observation period of 20 min. Their social behavior and interaction time was then scored as follows: number of social events (sniffing, following, grooming the partner, crawling over or under). The interaction time was defined as the time spent in active social interactions (sniffing, following, grooming the partner, wrestling, crawling over or under) [21, 97, 99]. The interaction time represents the total time spent in active behavior, regardless of the number of individual events. After each trial, animals were returned to their home cages and the arena was changed to a new and clean one, in order to avoid any odor cues from one pair to the next one.

### Statistical analysis

All experiments and analyses were carried out blindly with respect to genotypes until the codes were broken. All results are expressed as mean ± SEM. Significance was assessed with a Mann-Whitney non-parametric U test. Statistical analysis of biochemical measurements including two groups were performed using unpaired t-test or Mann-Whitney non-parametric U test or Welch's t-test (unequal variance). ANOVA (equal variance) followed by Tukey-Kramer or Newman-Keuls post hoc tests or with Welch's ANOVA (unequal variance) followed by a Dunnett's post hoc test were used to compare three groups. Significance of electrophysiological and morphological results was assessed with a Mann-Whitney non-parametric U test or one-way ANOVA followed by a protected Fisher post hoc test. All statistical analyses were performed using Origin 8.0 analysis software (OriginLab), JMP (version 9, SAS) or prism (version 4.0, GraphPad Software Inc.) and are presented in Table 1.

## Abbreviations

3xTg-AD: triple transgenic mouse model of Alzheimer's disease
Aβ: abeta peptide
AD: Alzheimer disease
AMPA: α-Amino-3-hydroxy-5-methyl-4-isoxazolepropionic acid
Cdk5: Cyclin-dependent kinase 5
dnPAK: dominant negative PAK
EPSCs: excitatory postsynaptic currents
GABA: gamma-aminobutyric acid
GAD65: glutamic acid decarboxylase 65 KDa
GluN2B: NMDA receptor GluN2B (GluR epsilon 2/NR2B) subunit
GSK3: glycogen synthase kinase 3
mEPSCs: miniature excitatory postsynaptic currents
mPSCs: miniature postsynaptic currents
NonTg: non-transgenic
PAK: p21-activated kinase
pPAK: phosphorylated PAK
PSCs: postsynaptic currents
PSD95: postsynaptic density protein 95
Ser: serine
Thr: threonine
TTX: tetradotoxin
VGAT: vesicular GABA transporter
